# Beating heart superior caval vein replacement for giant venous aneurysm: a case report

**DOI:** 10.1093/ehjcr/ytaf082

**Published:** 2025-02-14

**Authors:** Kira Osipenko, Daniel Zimpfer, Maria Anwar, Stephane Mahr, Paul Werner

**Affiliations:** Department of Cardiac and Thoracic Aortic Surgery, Medical University of Vienna, Spitalgasse 23, 1090 Vienna, Austria; Department of Cardiac and Thoracic Aortic Surgery, Medical University of Vienna, Spitalgasse 23, 1090 Vienna, Austria; Division of Cardiothoracic and Vascular Anesthesia and Intensive Care Medicine, Department of Anesthesia, Critical Care and Pain Medicine, Medical University of Vienna, Spitalgasse 23, 1090 Vienna, Austria; Department of Cardiac and Thoracic Aortic Surgery, Medical University of Vienna, Spitalgasse 23, 1090 Vienna, Austria; Department of Cardiac and Thoracic Aortic Surgery, Medical University of Vienna, Spitalgasse 23, 1090 Vienna, Austria

**Keywords:** Superior vena cava aneurysm, Cava replacement, Vein aneurysm, Case report

## Abstract

**Background:**

Superior vena cava (SVC) aneurysms are rare vascular anomalies, with <80 cases reported worldwide. Large aneurysms carry a risk of rupture and thrombosis, eventually necessitating surgical intervention.

**Case summary:**

We present the case of a 68-year-old male with a progressively enlarging SVC aneurysm and a history of paroxysmal atrial fibrillation. The patient underwent successful caval resection and replacement with a 20 mm expanded polytetrafluoroethylene (ePTFE) graft, accompanied by left atrial appendage exclusion and pulmonary vein isolation. The recovery from surgery was uneventful, and postoperative imaging confirmed excellent graft patency with no signs of thrombosis or recurrence at the three-month follow-up.

**Discussion:**

This case highlights the successful management of a large SVC aneurysm through complete resection and prosthetic replacement with a 20 mm ePTFE graft, with the first documented use of this technique in an adult patient. Caval resection and graft replacement ensure complete excision of affected tissue, minimizing the risk of recurrence after surgery for complex SVC aneurysms.

**Learning points**

- Superior caval aneurysms are a very rare clinical entity where individualized surgical planning is required.
- Caval resection and replacement with an expanded PTFE graft ensure complete removal of affected tissue, possibly reducing the risk of recurrence.

## Introduction

Superior vena cava aneurysms (SVCAs) are a rare clinical diagnosis with <80 cases described worldwide.^1^ While some propose a conservative strategy, surgical treatment has been predominantly performed, especially in cases of aneurysm growth, because large aneurysms present a risk of rupture and thrombosis, eventually requiring surgical intervention.^2^ We herein describe to our knowledge the first case of caval replacement with an ePTFE prosthesis as a possible surgical strategy for SVCAs.

## Summary figure

**Figure:**
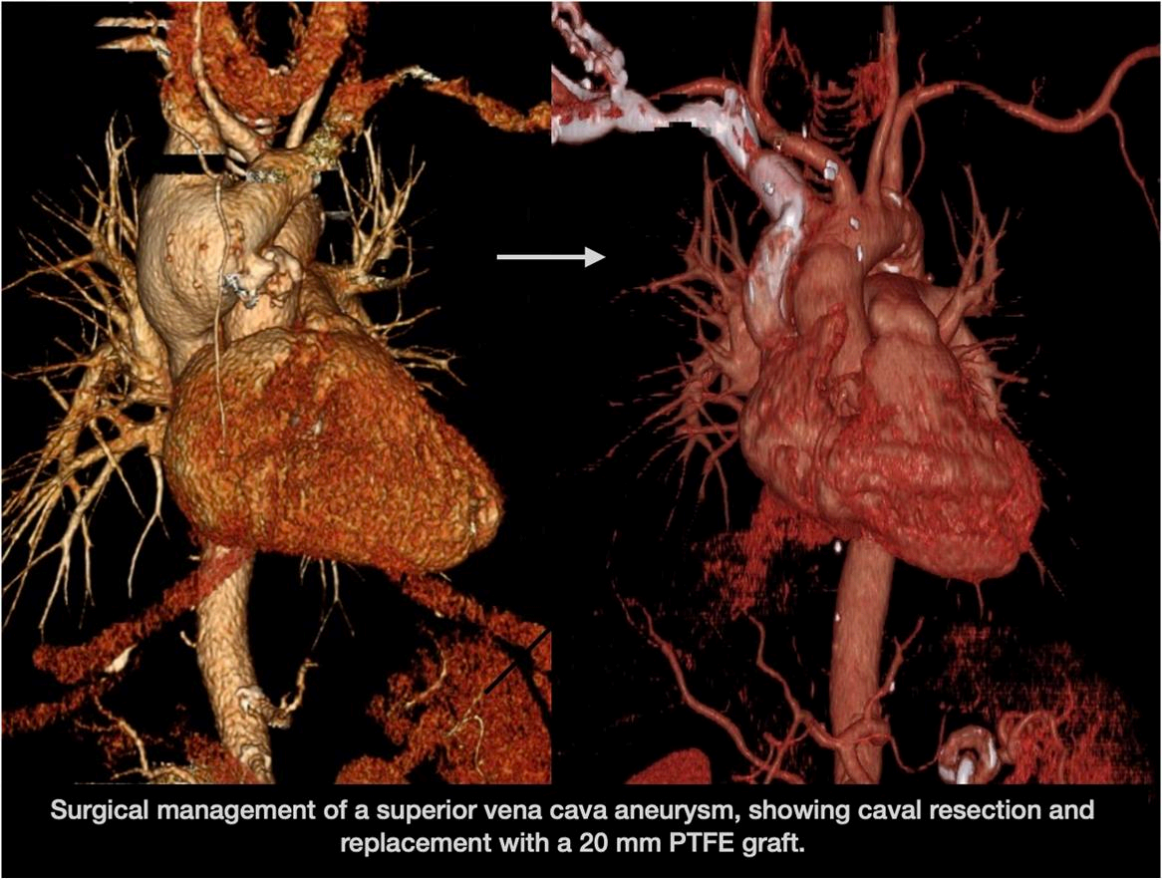


## Case presentation

**Figure 1:**
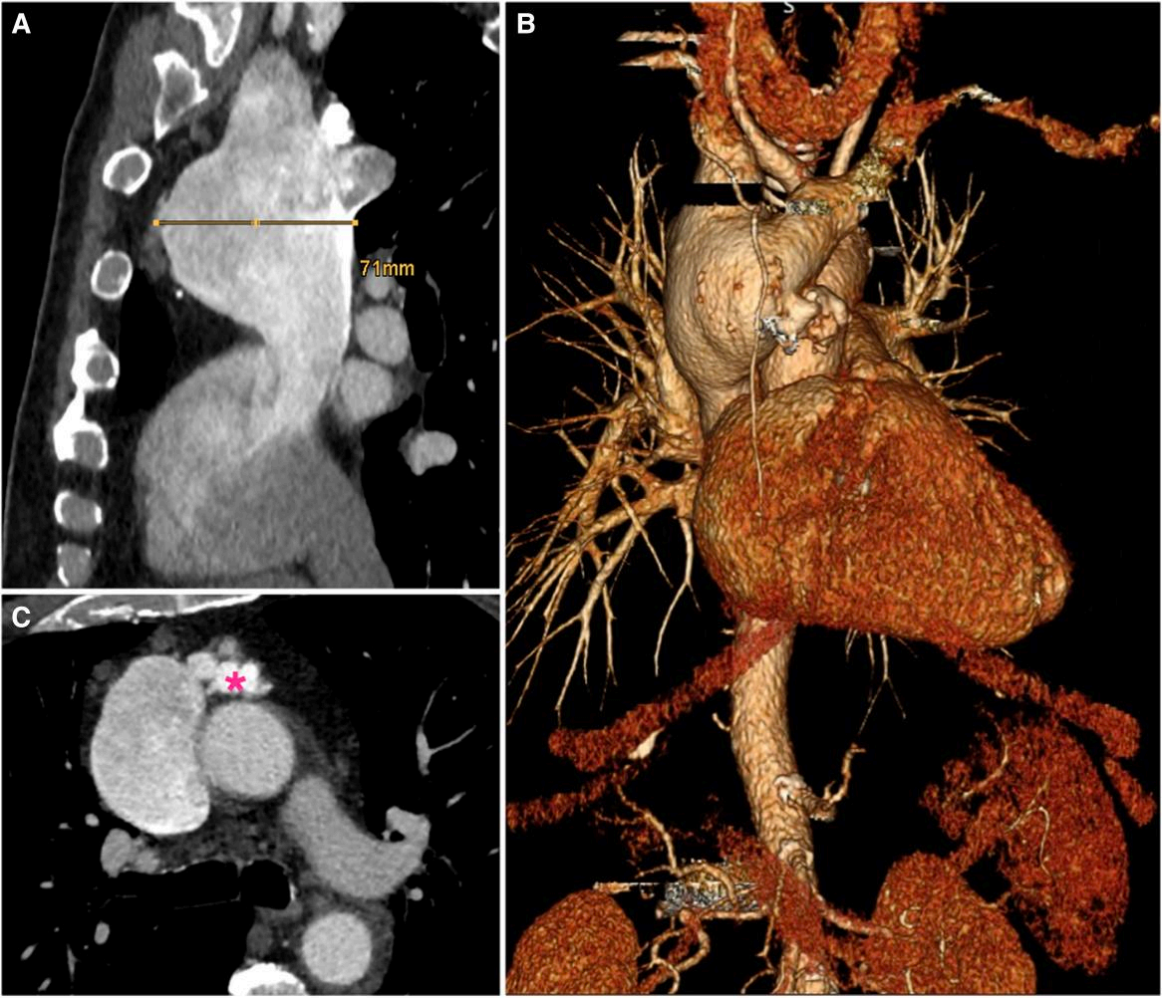
Preoperative CTA illustrating the aneurysm of the superior vena cava (SVC). (*A*) 7 cm maximum diameter in anterior–posterior axis. (*B*) 3D reconstruction of the SVC aneurysm. (*C*) Atypical venous malformations (asterisk) below the brachiocephalic vein origin.

A 68-year-old asymptomatic male patient was incidentally diagnosed with a fusiform SVCA on computed tomographic angiography (CTA) performed for further evaluation of a suspicious mass seen on a chest X-ray. He was followed up for two years at our outpatient clinic, and a disease progression was notable. At last follow-up, the venous aneurysm measured at 7 cm involving the innominate and right subclavian veins (*Figure 1*). Due to the presence of atypical venous malformations and to rule out a possible malignancy, a positron emission tomography-magnetic resonance imaging was performed, showing no suspicious fluorodeoxyglucose uptake. The patient also suffered from paroxysmal atrial fibrillation and had a history of two ablation procedures for atrioventricular nodal re-entrant tachycardia. Coronary angiography demonstrated no significant coronary artery disease, and transthoracic echocardiography revealed good biventricular function and unremarkable valve status. The physical examination, including heart auscultation, showed no abnormalities.

Surgical correction of the SVCA with concomitant atrial fibrillation ablation and left atrial appendage exclusion was planned.

### Perioperative course

Following median sternotomy, dissection of the innominate vein, the right subclavian vein, the right jugular vein, and the mostly extra-pericardial and extra-pleural located SVCA was performed with care to the surrounding structures such as the right phrenic and recurrent nerve.

Following full heparinization, arterial cannulation was performed in the ascending aorta and venous cannulation directly in the inferior vena cava with a 32 French angled tip and percutaneously in the right jugular vein via a 17 French heparin-coated cannula in Seldinger technique. After initiation of cardiopulmonary bypass (CPB), radiofrequency ablation of the pulmonary veins was performed and a 40 mm AtriClip (Atricure, Mason, OH, USA) was applied to the left atrial appendage. An additional venous cannula (20 mm metal tip) was introduced in the innominate vein, and total CPB was initiated.

**Figure 2:**
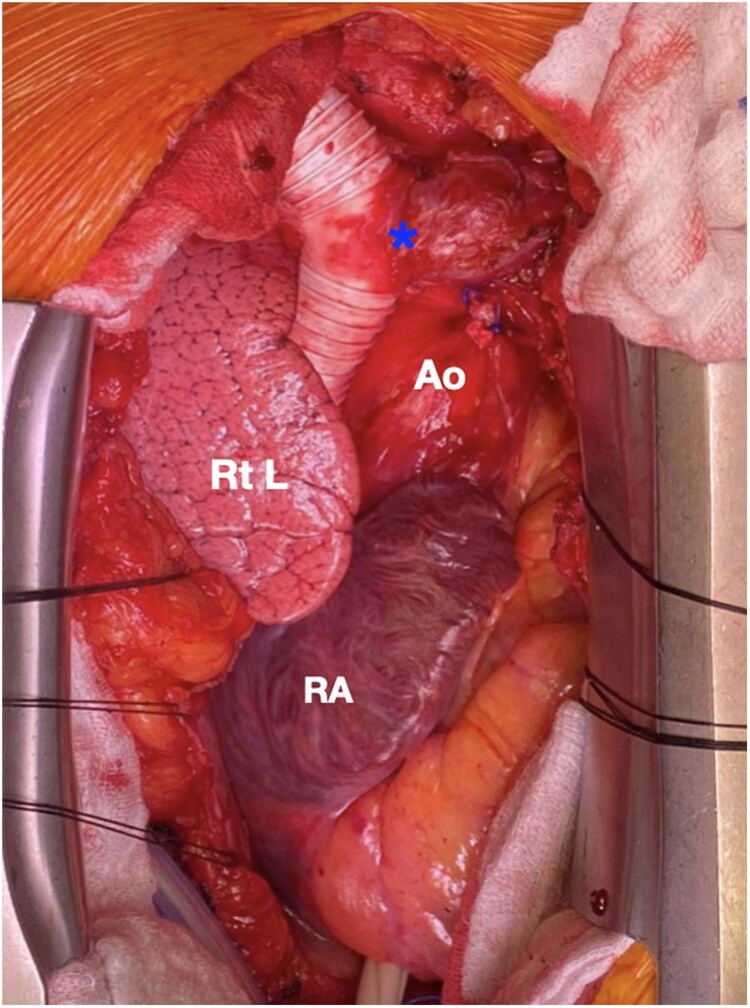
Intraoperative image demonstrating the operative result after 20 mm PTEF graft replacement of the superior vena cava, with the asterisk indicating the end-to-side anastomosis of the brachiocephalic vein. Ao, ascending aorta; Rt L, right lung; RA, right atrium.

The venous aneurysm was completely resected up to the origin of the brachiocephalic vein, while the azygos vein was ligated externally. The aneurysm was sent for frozen section procedure, where no malignancy was found, thus the phrenic nerve could be spared. The distal anastomosis to the right subclavian vein was performed with a 20 mm PTFE graft and 5-0 polypropylene in running technique. The brachiocephalic vein was anastomosed end-to-side with that 20 mm graft, and the proximal anastomosis of the graft was then sewn to the cava stump at the right atrium (*Figure 2*). Uneventful cardiopulmonary bypass weaning and subsequent chest closure concluded the procedure.

Following extubation and catecholamine weaning, the patient was transferred to the normal ward on the second postoperative day. A left-sided vocal cord motility disorder was noted before discharge, which fully resolved after one month of treatment with medications containing vitamins B1 and B6.

**Figure 3:**
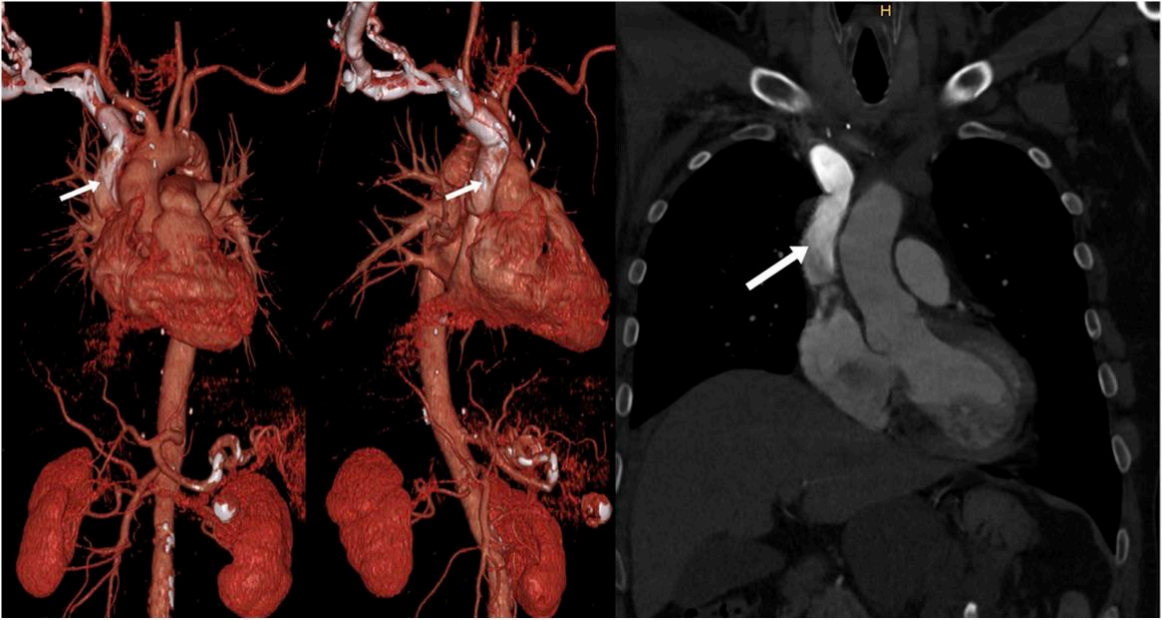
Postoperative CTA at 3-month follow-up demonstrated a satisfactory surgical result with homogenous contrast filling of the PTFE graft without any signs of thrombosis (white arrow).

At three-month follow-up, the patient showed excellent recovery, and CTA demonstrated no pathological findings with good contrast filling of the PTFE graft without any signs of thrombosis (*Figure 3*). Due to recurrent episodes of atrial fibrillation, the patient was placed on anticoagulation with rivaroxaban.

## Discussion

Superior vena cava aneurysms are extremely rare vascular abnormalities, generally classified into two subtypes: fusiform and saccular.

Fusiform aneurysms are the more frequently encountered variant and are typically identified incidentally, as most patients remain asymptomatic.^3^ These aneurysms are generally associated with a benign clinical course and are managed conservatively, with anticoagulation therapy and regular monitoring to prevent complications.^1,4^

Saccular aneurysms, on the other hand, are considerably less common. They typically develop as a consequence of post-interventional or post-inflammatory events and are associated with a significantly elevated risk of serious complications, such as rupture, thrombosis, and venous obstruction. Due to these risks, surgical resection is generally the treatment of choice, particularly when there are concerns about the aneurysm’s morphology, its increasing size beyond 40 mm, or the presence of thrombus.^1,5^

Various surgical approaches for the treatment of SVCAs have been described in the literature. In early reports from the 1950s, one case described treating an aneurysm by applying polythene cellophane to the affected area.^6^ Nowadays, the most common approach for managing SVCAs is caval reconstruction using a bovine pericardial patch. Although easily reproducible, this technique presents with a shortcoming, that not all aneurysmatic tissue is resected. In contrast, when performing caval resection and replacement as described in this case, complete resection of affected tissue limits the chance of recurrence. An expanded polytetrafluoroethylene graft was the conduit of choice as it demonstrates good long-term durability with lower dilatation rates, particularly in comparison to polyester grafts.^7^ Due to their longevity, ePTFE grafts are commonly used as venous conduits in paediatric cardiac surgery, namely the extracardiac Fontan procedure, which demonstrates favourable long-term outcomes with a 32-year survival probability of 84% and a graft failure risk of 12%.^8^ However, long-term studies also mentioned potential risks, such as stenosis and thrombosis and the necessity of anticoagulation.^9^ Currently, no data are available on the long-term outcomes of ePTFE grafts for superior vena cava replacement, particularly regarding the risks of thrombosis or stenosis.

For high-risk patients where surgery is not feasible, endovascular techniques may be employed. One such approach is the first reported use of balloon-protected transcatheter thrombin injection, applying latex balloon protection to avoid SVC thrombosis.^10^ This technique could be a valuable alternative for treating large and growing saccular central vein aneurysms in this patient group. The application in fusiform aneurysms, however, might be of limited value. A recently described true transcatheter alternative includes coil occlusion of the vein, placement of an uncovered stent in the SVC, and coil closure of the aneurysm sac’s neck through the stent framework.^5^

## Conclusion

Superior vena cava aneurysms present a rare clinical entity, with surgical (and endovascular) approaches available depending on the patient’s risk profile. While conservative management may be applied in absence of aneurysmal growth, our case highlights the necessity of timely surgical intervention. The use of a 20 mm PTFE graft for reconstruction demonstrates mirrors established paediatric cardiovascular procedures and provides a novel solution for adult SVCAs.

## Data Availability

Patient data will not be shared.

## Lead author biography

Kira Osipenko, MD, graduated from the Medical University in 2023 and is currently pursuing a PhD at the Medical University of Vienna, working at the Department of Cardiac and Thoracic Aortic Surgery at Vienna General Hospital. Her research focuses on valve and aneurysm surgery, with a particular interest in transcatheter aortic valve implantation and minimally invasive procedures. She is actively involved in advancing techniques in these areas and contributing to clinical research in cardiac surgery.

**Consent:** In line with COPE (Committee on Publication Ethics) best practice guidelines, patient consent for publication has been obtained.

**Funding:** The authors received no financial support for the research, authorship, and/or publication of this article.
